# Weakness Status is Differentially Associated with Time to Diabetes in Americans

**DOI:** 10.20900/agmr20240004

**Published:** 2024-07-24

**Authors:** Kelly Knoll, Yeong Rhee, Natasha Fillmore, Donald A. Jurivich, Justin J. Lang, Brenda M. McGrath, Grant R. Tomkinson, Ryan McGrath

**Affiliations:** 1 Department of Health, Nutrition, and Exercise Sciences, North Dakota State University, Fargo, ND 58108, USA; 2 Healthy Aging North Dakota, North Dakota State University, Fargo, ND 58102, USA; 3 Department of Pharmaceutical Sciences, North Dakota State University, Fargo, ND 58108, USA; 4 Department of Geriatrics, University of North Dakota, Grand Forks, ND 58202, USA; 5 Centre for Surveillance and Applied Research, Public Health Agency of Canada, Ottawa, ON K1A 0K9, Canada; 6 School of Epidemiology and Public Health, University of Ottawa, Ottawa, ON K1N 6N5, Canada; 7 Alliance for Research in Exercise, Nutrition and Activity (ARENA), Allied Health and Human Performance, University of South Australia, Adelaide, SA 5000, Australia; 8 OCHIN Inc., Portland, OR 97228, USA; 9 Fargo VA Healthcare System, Fargo, ND 58102, USA

**Keywords:** aging, mass screening, muscle strength, muscle strength dynamometer

## Abstract

**Background:**

The purpose of this study was to evaluate the associations of (1) individual absolute and body size normalized weakness cut-points, and (2) the collective weakness classifications on time to diabetes in Americans.

**Methods:**

We analyzed data from 9577 adults aged at least 50-years from the Health and Retirement Study. Diabetes diagnosis was self-reported. A handgrip dynamometer measured handgrip strength (HGS). Males with HGS <35.5 kg (absolute), <0.45 kg/kg (normalized to body weight), or <1.05 kg/kg/m^2^ (normalized to BMI) were categorized as weak. Females were classified as weak if their HGS was <20.0 kg, <0.337 kg/kg, or <0.79 kg/kg/m^2^. Compounding weakness included falling below 1, 2, or all 3 cut-points.

**Results:**

Persons below the body weight normalized weakness cut-points had a 1.29 (95% confidence interval (CI): 1.15–1.47) higher hazard for incident diabetes, while those below the BMI normalized cut-points had a 1.30 (CI: 1.13–1.51) higher hazard. The association between absolute weakness and incident diabetes was insignificant (hazard ratio: 1.06; CI: 0.91–1.24). Americans below 1, 2, or all 3 collective weakness categories had a 1.28 (CI: 1.10–1.50), 1.29 (CI: 1.08–1.52), and 1.33 (CI: 1.09–1.63) higher hazard for the incidence of diabetes, respectively.

**Conclusions:**

Our findings indicate that while absolute weakness, which is confounded by body size, was not associated with time to diabetes, adjusting for the influence of body size by normalizing HGS to body weight and BMI was significantly associated with time to diabetes. This suggests that muscle strength, not body size, may be driving such associations with time to diabetes.

## INTRODUCTION

Approximately 15% of middle-aged and 25% of older adults in the United States have been diagnosed with diabetes [1]. The presence of diabetes increases risk for multiple long-term chronic conditions, functional disability, and early all-cause mortality [2,3]. Physical inactivity and diet are hallmark risk factors for diabetes [4,5]. Sedentary behavior and diet are also linked to strength capacity [6,7], and despite cardiorespiratory fitness being a long-standing risk factor for diabetes [8], muscle strength is associated with diabetes independent of estimated cardiorespiratory fitness [9]. As such, routine measurement of strength capacity by healthcare providers may help with early screening, referral to intervention, and diabetes prevention.

Handgrip strength (HGS) is a convenient and reliable measure of overall muscle strength that is used in clinical, translational research, and population-based settings [10,11]. Low handgrip strength is associated with pre-diabetes and diabetes [12,13]. Weakness, as measured by HGS, is often clinically defined when HGS is below a pre-specified threshold (i.e., cut-point). Several cut-points for weakness exist that are often anchored to slow gait speed (physical disablement) [14], but other thresholds are specified to diabetes [15-17]. However, the Sarcopenia Definition and Outcomes Consortium (SDOC) recently generated weakness cut-points that include absolute HGS, and measures of HGS that are normalized to body weight and body mass index (BMI) [18-20]. Given that body size is related to muscle strength and may improve the precision of HGS measurements as a stand-alone metric of strength capacity, normalizing HGS to body size may help improve criteria for diagnosing clinically-relevant health conditions related to diabetes such as sarcopenic obesity [21,22].

Although the SDOC has created weakness classifications for absolute and normalized HGS, the collective use of these thresholds may help to improve the operationalization of weakness and their predictive value. For example, previous work has revealed that the individual and collective use of the SDOC weakness cut-points were differentially associated with cognitive impairment [23], which is not directly part of the physical disablement cascade [24]. Therefore, examining the individual and collective use of the SDOC weakness cut-points for diabetes may provide insights for HGS as a screening tool for chronic cardiometabolic diseases. The purpose of this study was to evaluate the associations between each absolute and body size normalized weakness cut-point on time to diabetes in Americans. We also sought to determine the associations between the collective weakness categories on incident diabetes in Americans.

## MATERIALS AND METHODS

### Participants

A secondary analysis of data from those aged at least 50-years from the 2006–2018 waves of the RAND Health and Retirement Study (HRS) was conducted. The HRS uses an observational design which includes a longitudinal-panel structure, wherein participants enter the HRS, complete core interviews biennially, and are followed until death for observing health factors in Americans during aging [25]. The HRS maintains a national sample by occasionally adding new cohort samples [26]. Interview response rates have routinely been 80%–90% [27]. More details about the HRS are available elsewhere [28].

Live, in-person interviews were incorporated in the HRS starting with the 2006 wave, which included physical measures such as HGS [25]. Such in-person interviews were executed on random half sub-samples of HRS participants, with these interviews occurring every other wave, while the other half sub-sample only completed the core interviews so that overall study burden could be reduced [25]. All participants provided written informed consent before entering the HRS, and the University of Michigan Health Sciences/Behavioral Sciences Institutional Review Board approved protocols (HUM00061128).

### Measures

#### Diabetes

Respondents told interviewers if a healthcare provider had ever diagnosed them with diabetes. Persons self-reporting a diagnosis after their baseline interview were considered as having diabetes.

#### Handgrip strength

HGS was collected with a Smedley spring-type handgrip dynamometer (Scandidact; Odder, Denmark) [29]. Participants were eligible for HGS assessments if they did not report having surgery, swelling, severe pain, or an injury to both hands at least 6-months prior to the interview. If HGS assessment criteria limited HGS on a single hand then only the other hand was examined. HRS interviewers fitted the handgrip dynamometer to the hand size of each participant, and allowed for a practice trial on the reported dominant hand. Participants were instructed to remain standing while grasping the dynamometer with their arm by their side at a 90° angle. Protocol alternatives were allowed for those unable to stand or grasp the dynamometer appropriately wherein participants could sit and rest their arm on a supporting object. The handgrip dynamometer was placed in the non-dominant hand to begin testing, and participants squeezed the dynamometer as hard as possible for two alternating trials on each hand. There was a brief rest period between trials if HGS was only tested on one hand Additional details about the HGS protocols in the HRS are available elsewhere [29].

The highest recorded HGS value irrespective of hand tested was included in the analyses. Body weight was self-reported, and BMI was calculated from reported standing height and body weight as kilograms per meters-squared. For the absolute and body size normalized cut-points, males were categorized as weak if their HGS was <35.5 kg (absolute), <0.45 kg/kg (normalized to body weight), or <1.05 kg/kg/m^2^ (normalized to BMI), while females were considered weak if their HGS was <20.0 kg, <0.337 kg/kg, or <0.79 kg/kg/m^2^ [18-20]. Collective weakness categorized participants as being below 1, 2, or all 3 cut-points.

#### Covariates

Participants self-reported their age, sex, race, standing height, and body weight. Participants similarly reported if a healthcare provider had ever diagnosed them with hypertension, stroke, or arthritis (or rheumatism). Current and previous cigarette smoking (100 cigarettes smoked in their lifetime) status was self-reported. A one-item perceived health indicator asked participants to score their health status as “excellent”, “very good”, “good”, “fair”, or “poor”. Interviewers asked respondents about their abilities to perform activities of daily living (ADL): dress, eat, transfer in-or-out of bed, toilet, bathe, and walk across a small room. Tasks included in the ADL assessment from the HRS are generally modeled from the Katz Index. Those signifying difficulty or an inability to complete ≥1ADL task were considered as having a basic self-care limitation.

Depressive symptomology was evaluated with the 8-item Center for the Epidemiologic Studies Depression scale [30]. Participants reported within the week prior to the interview if they experienced any positive or negative emotions. Participants with scores ≥3 were considered as depressed [30]. Cognitive function was ascertained with the modified version of the Telephone Interview of Cognitive Status, which was created from the Mini-Mental State Examination for epidemiological studies such as the HRS. Examinations in the modified Telephone Interview of Cognitive Status reflect neurophysiological health, which includes attributes such as recall and executive function. Those with scores ≤10 were considered as having a cognitive impairment. Participants reporting involvement in moderate-to-vigorous physical activity (MVPA) at least “once a week” were classified as participating in MVPA [31].

### Statistical Analysis

All analyses were performed with SAS 9.4 software (SAS Institute; Cary, NC). Baseline descriptive characteristics of the participants were presented as mean ± standard deviation for continuous variables and frequency (percentage) for categorical variables. Individual Cox proportional hazard regression models quantified the associations of (1) absolute weakness (reference: not-below absolute cut-point), (2) body weight normalized weakness (reference: not-below the body weight normalized cut-point), and (3) BMI normalized weakness (reference: not-below the BMI normalized cut-point), and (4) collective weakness (reference: below 0 cut-points) on time to diabetes. From each model, hazard ratios were generated to assess the prospective association between the measure of weakness and the risk of newly reported diabetes diagnosis. The Cox models were adjusted for age, sex, race, hypertension, stroke, arthritis, cigarette smoking status, self-rated health, depression, MVPA participation, cognitive impairment, and ADL limitations.

Data were left-truncated because HRS participants entered at different ages and had to be aged at least 50-years to be included. Participants enter the HRS at different ages because the HRS utilizes an observational design (i.e., longitudinal-panel), but persons that may have entered the HRS after the origin point (i.e., ≥50-years) have a delayed entry into the observation (i.e., left-truncation) [32]. Therefore, age at baseline was the entry variable. We selected a time to event (i.e., diabetes) analysis, which thereby excluded persons with diabetes at baseline, because the presence of diabetes is linked to diminished strength capacity from factors such as diabetic peripheral neuropathy [33].

As a supplementary analysis, we utilized the same Cox model procedures for determining the association of persons below alternate, but diabetes specific body weight normalized weakness cut-points (0.68 kg/kg for males and 0.49 kg/kg in females) [16] and incident diabetes. Further, we used the Cox model procedures from our principal analyses for evaluating the associations of the individual and collective weakness categories on time to diabetes by sex. We also conducted a Cox model with these procedures for quantifying the associations between all possible weakness category permutations and time to diabetes. An alpha level of 0.05 was used for all analyses.

## RESULTS

The descriptive characteristics of the participants are in Table 1. Overall, participants were aged 68.2 ± 10.8 years and mostly female (56.7%). The results for the associations of the specific weakness categories on time to diabetes are shown in Table 2. Participants below the BMI normalized weakness cut-point had a 1.30 (95% confidence interval (CI): 1.13, 1.51) higher hazard for diabetes, while those below the body weight normalized cut-point had a 1.29 (CI: 1.15, 1.47) higher hazard for diabetes. Table 3 reveals the results for the associations of the collective weakness categories on time to diabetes. Persons below each collective weakness cut-point had a higher hazard ratio for incident diabetes: 1.28 (CI: 1.10, 1.50) for 1 cut-point, 1.29 (CI: 1.08, 1.52) for 2 cut-points, and 1.33 (CI: 1.09, 1.63) for all 3 weakness cut-points.

Appendix A1 presents the results for the association between the alternate body weight normalized weakness cut-points on time to diabetes. Persons below these body weight normalized weakness cut-points did not have a higher hazard for incident diabetes (hazard ratio: 1.32; CI: 0.98, 1.78). Appendix A2 shows the results for the associations of the individual weakness categories on time to diabetes by sex. Males below the BMI and body weight normalized weakness cut-points had a 1.53 (CI: 1.17, 2.00) and 1.37 (CI: 1.14, 1.65) higher hazard for diabetes, respectively. Females similarly below the BMI and body weight normalized weakness cut-points had a 1.22 (CI: 1.02, 1.45) and 1.26 (CI: 1.06, 1.50) higher hazard for diabetes, respectively. No significant associations were observed for persons below the absolute weakness category and time to diabetes for males and females. The results for the associations of the compounding weakness categories on time to diabetes by sex are in Appendix A3. In males, those below 1 weakness category had a 1.36 (CI: 1.10, 1.68) higher hazard for diabetes, while males below 3 weakness categories had a 1.65 (CI: 1.20, 2.26) higher hazard. In females, only those below 2 weakness categories had a 1.33 (CI: 1.07, 1.64) higher hazard for diabetes. Appendix A4 presents the results for all possible weakness category permutations on time to diabetes. Significant associations were observed for participants with body weight normalized weakness (hazard ratio: 1.31; CI: 1.11, 1.54), BMI and body weight normalized weakness (hazard ratio: 1.45; CI: 1.20, 1.76), and all possible weakness categories (hazard ratio: 1.33; CI: 1.09, 1.63).

## DISCUSSION

The principal findings of our investigation showed that weakness was associated with time to diabetes in Americans. Specifically, persons below the body weight and BMI normalized weakness cut-points had 29% and 30% greater risk for diabetes, respectively. However, no statistically significant associations were observed for diabetes regarding those beneath the absolute weakness threshold. Persons below 1, 2, or all 3 collective weakness categories were also at greater risk for diabetes, but the magnitude of the hazard ratios were similar across the collective weakness groups. Our findings indicate that absolute weakness, which is confounded by body size [34], is not associated with time to diabetes, but removing the confounding influence of body size on strength capacity by normalizing HGS to body weight and BMI revealed an association with time to diabetes. This suggests that strength capacity, not body size, could be driving such associations with diabetes.

Our results conflict with another investigation evaluating the SDOC weakness cut-points individually and collectively for cognitive function, which found that only older Americans below the absolute weakness cut-point had greater odds for future cognitive impairment [23], Alternatively, our findings align with other studies that showed body weight normalized weakness was associated with diabetes [15,17], and the body weight normalized weakness cut-points generated from these investigations were similar to the thresholds used in our study [18-20]. However, our supplementary findings did not show a statistically significant association for other body weight normalized weakness cut-points and incident diabetes [16], likely because these cut-points were higher than those from the SDOC [18-20]. These findings further illustrate how different weakness cut-points may influence comparisons of findings across investigations including HGS [14].

The results from our investigation emphasize the importance of strength capacity as a risk factor for diabetes. Indicators of insulin sensitivity, such as muscle fiber type and muscle mass, may help to explain the association between weakness and time to diabetes [35,36]. Healthcare providers are encouraged to regularly assess muscle strength as measured with HGS with their patients and converse about the importance of strength capacity with relevant patients. Primary interventions for diabetes, worksite health promotion platforms [37], and public health initiatives such as the diabetes prevention program [38] may also benefit from assessing HGS, as strength capacity could be a summative index of several bodily systems that reflect physical activity profiles. Future research should leverage implementation science framework for incorporating HGS measurements into healthcare settings as applicable [39,40], and consider the role of genetics for strength capacity.

## LIMITATIONS

Some limitations should be acknowledged. Participants may have had undiagnosed or delayed diagnoses of diabetes, which may have led to underestimations for our findings. Although diabetes diagnosis was self-reported in our study, publicly available HRS data do not include blood biomarkers for objectively diagnosing diabetes. Other covariates included in our analyses were also self-reported. Since HRS core interviews occurred every 2-years, a report of new diabetes diagnosis may have lacked time precision from when diabetes was clinically diagnosed (i.e., diagnosis may have occurred between 2-year waves). While the HRS is a large population-based study, multiple trained interviewers collecting data from participants may have threatened internal validity. Factors that may have influenced the association between weakness and time to diabetes may not have been available in the HRS (e.g., specificity and duration of medication usage, diet logs). The SDOC cut-points used in our study did not normalize for other body size metrics such as stature [41].

## CONCLUSIONS

This investigation found that Americans below the body weight and BMI normalized weakness cut-points from the SDOC were at greater risk for incident diabetes, but this was not the case for persons beneath the absolute weakness cut-points. Low strength capacity could be driving these associations between weakness and time to diabetes given the confounding effect of body size on the absolute cut-points. Those in each collective weakness category also had an increased risk of incidence for diabetes, with the magnitude of risk being similar across groups indicating no additional predictive benefit for diabetes. Nevertheless, HGS is recommended as a feasible measure of strength capacity and screening tool for future diabetes.

## Figures and Tables

**Table 1.** Baseline descriptive characteristics of the participants.

Variable	Overall (*n* = 9577)	No Diabetes (*n* = 8421)	Developed Diabetes (*n* = 1156)
Age (years)	68.2 ± 10.8	68.4 ± 11.0	67.3 ± 9.3
Body Mass Index (kg/m^2^)	27.6 ± 5.4	27.3 ± 5.3	30.0 ± 5.6
Female (*n* (%))	5432 (56.7)	4809 (57.1)	623 (53.8)
White (*n* (%))	7289 (76.1)	6478 (76.9)	811 (70.1)
Hypertension (*n* (%))	5358 (55.9)	4593 (54.5)	765 (66.1)
Stroke (*n* (%))	646 (6.7)	564 (6.7)	82 (7.0)
Arthritis (*n* (%))	5501 (57.4)	4795 (56.9)	706 (61.0)
Cigarette Smoking Status (*n* (%))			
Current Smoker	1398 (14.6)	1248 (14.8)	150 (13.0)
Previous Smoker	3966 (41.4)	3454 (41.0)	512 (44.3)
Never Smoked	4213 (44.0)	3719 (44.2)	494 (42.7)
Self-Rated Health (*n* (%))			
Excellent	1146 (12.0)	1039 (12.3)	107 (9.2)
Very Good	3122 (32.6)	2794 (33.1)	328 (28.3)
Good	3091 (32.3)	2686 (31.9)	405 (35.0)
Fair	1752 (18.3)	1506 (17.8)	246 (21.2)
Poor	466 (4.8)	396 (4.7)	70 (6.0)
Depressed (*n* (%))	1182 (12.3)	1021 (12.1)	161 (13.9)
MVPA Participation (*n* (%))	5830 (60.8)	5171 (61.4)	659 (57.0)
ADL Limitation (*n* (%))	1278 (13.3)	1085 (12.8)	193 (16.7)
Cognitive Impairment (*n* (%))	149 (1.5)	132 (1.5)	17 (1.4)

Note: ADL = activities of daily living, MVPA = moderate-to-vigorous physical activity.

**Table 2.** Results for the associations of the specific weakness categories on time to diabetes.

Weakness Category	Participants	Diabetes Cases	Average & 95% CI Follow-Up Years	Diabetes Rate per 1000 Person-Years	Hazard Ratio (95% CI)
Absolute					
Not-Weak	7304 (76.3%)	938 (12.8%)	6.8 (6.7, 6.9)	18.8	Reference
Weak	2273 (23.7%)	218 (9.6%)	6.3 (6.2, 6.5)	15.1	1.06 (0.91, 1.24)
BMI Normalized					
Not-Weak	7352 (78.8%)	858 (11.7%)	6.8 (6.7, 6.9)	17.1	Reference
Weak	2225 (23.3%)	298 (13.4%)	6.4 (6.3, 6.4)	20.9	1.30 (1.13, 1.51)
Body Weight Normalized					
Not-Weak	5353 (55.9%)	579 (10.8%)	6.9 (6.8, 7.0)	15.6	Reference
Weak	4224 (44.1%)	577 (13.7%)	6.4 (6.3, 6.6)	21.2	1.29 (1.15, 1.47)

Note: BMI = body mass index, CI = confidence interval.

**Table 3.** Results for the associations of the collective weakness categories on time to diabetes.

Weakness Category	Participants	Diabetes Cases	Average & 95% CI Follow-Up Years	Diabetes Rate per 1000 Person-Years	Hazard Ratio (95% CI)
Overall					
0 Weakness Categories	5,013 (52.3%)	558 (11.1%)	7.0 (6.9, 7.1)	16.0	Reference
1 Weakness Category	1705 (17.8%)	243 (14.3%)	6.5 (6.3, 6.6)	22.0	1.28 (1.10, 1.50)
2 Weakness Categories	1560 (16.3%)	215 (13.8%)	6.4 (6.2, 6.6)	21.5	1.29 (1.08, 1.52)
3 Weakness Categories	1299 (13.6%)	140 (10.8%)	6.3 (6.1, 6.6)	16.9	1.33 (1.09, 1.63)

Note: CI = confidence interval.

## Data Availability

The dataset generated from (or analyzed in) the study can be found at the Health and Retirement website: https://hrs.isr.umich.edu/data-products.

## APPENDICES

**Appendix A1.** Hazard ratios for the associations of the alternate body weight normalized weakness category on time to diabetes.

Weakness Category	Hazard Ratio (95% CI)
Not-Weak	Reference
Weak	1.32 (0.98, 1.78)

Note: CI = confidence interval.

**Appendix A2.** Hazard ratios for the associations of the specific weakness classifications on time to diabetes by sex.

Weakness Category	Hazard Ratio (95% CI)
**Males**	
Absolute	
Not-Weak	Reference
Weak	1.08 (0.87, 1.36)
BMI Normalized	
Not-Weak	Reference
Weak	1.53 (1.17, 2.00)
Body Weight Normalized	
Not-Weak	Reference
Weak	1.37 (1.14, 1.65)
**Females**	
Absolute	
Not-Weak	Reference
Weak	1.04 (0.83, 1.31)
BMI Normalized	
Not-Weak	Reference
Weak	1.22 (1.02, 1.45)
Body Weight Normalized	
Not-Weak	Reference
Weak	1.26 (1.06, 1.50)

Note: BMI = body mass index, CI = confidence interval.

**Appendix A3.** Hazard ratios for the associations of the compounding weakness categories on time to diabetes by sex.

Weakness Category	Hazard Ratio (95% CI)
Males	
0 Weakness Categories	Reference
1 Weakness Category	1.36 (1.10, 1.68)
2 Weakness Categories	1.19 (0.90, 1.57)
3 Weakness Categories	1.65 (1.20, 2.26)
Females	
0 Weakness Categories	Reference
1 Weakness Category	1.25 (0.99, 1.59)
2 Weakness Categories	1.33 (1.07, 1.64)
3 Weakness Categories	1.21 (0.93, 1.56)

Note: CI = confidence interval.

**Appendix A4.** Hazard ratios for the associations of the weakness category permutations on time to diabetes.

Weakness Category	Hazard Ratio (95% CI)
Not-Weak	Reference
Body Weight Normalized Weakness	1.31 (1.11, 1.54)
BMI Normalized Weakness	1.62 (0.52, 5.11)
Absolute Weakness	0.97 (0.58, 1.60)
BMI + Body Weight Normalized Weakness	1.45 (1.20, 1.76)
Absolute + Body Weight Normalized Weakness	1.01 (0.76, 1.33)
Absolute + BMI Normalized Weakness	3.09 (0.76, 12.58)
Absolute + BMI Normalized + Body Weight Weakness	1.33 (1.09, 1.63)

Note: CI=confidence interval.

## ABBREVIATIONS

ADL: activities of daily living
BMI: body mass index
CI: confidence interval
HGS: handgrip strength
HRS: Health and Retirement Study
MVPA: moderate-to-vigorous physical activity
SDOC: Sarcopenia Definition and Outcomes Consortium

## References

[1] CDC. National Diabetes Statistics Report. Available from: https://www.cdc.gov/diabetes/php/data-research/?CDC_AAref_Val=https://www.cdc.gov/diabetes/data/statistics-report/index.html. Accessed 2024 Jun 18.

[2] Ong KL, Stafford LK, McLaughlin SA, Boyko EJ, Vollset SE, Smith AE, Global, regional, and national burden of diabetes from 1990 to 2021, with projections of prevalence to 2050: a systematic analysis for the Global Burden of Disease Study 2021. Lancet. 2023;402(10397):203–34. 37356446 10.1016/S0140-6736(23)01301-6 PMC10364581

[3] Khunti K, Chudasama YV, Gregg EW, Kamkuemah M, Misra S, Suls J, Diabetes and Multiple Long-term Conditions: A Review of Our Current Global Health Challenge. Diabetes Care. 2023;46(12):2092–101. 38011523 10.2337/dci23-0035 PMC10698221

[4] O’Hearn M, Lara-Castor L, Cudhea F, Miller V, Reedy J, Shi P, Incident type 2 diabetes attributable to suboptimal diet in 184 countries. Nat Med. 2023;29(4):982–95. 37069363 10.1038/s41591-023-02278-8 PMC10115653

[5] CDC. Diabetes Risk Factors. Available from: https://www.cdc.gov/diabetes/risk-factors/?CDC_AAref_Val=https://www.cdc.gov/diabetes/basics/risk-factors.html. Accessed 2024 Jun 18.

[6] Ramsey KA, Rojer AG, D’Andrea L, Otten RH, Heymans MW, Trappenburg MC, The association of objectively measured physical activity and sedentary behavior with skeletal muscle strength and muscle power in older adults: A systematic review and meta-analysis. Ageing Res Rev. 2021;67:101266. 33607291 10.1016/j.arr.2021.101266

[7] Cruz-Jentoft AJ, Hughes BD, Scott D, Sanders KM, Rizzoli R. Nutritional strategies for maintaining muscle mass and strength from middle age to later life: A narrative review. Maturitas. 2020;132:57–64. 31883664 10.1016/j.maturitas.2019.11.007

[8] Cai L, Gonzales T, Wheeler E, Kerrison ND, Day FR, Langenberg C, Causal associations between cardiorespiratory fitness and type 2 diabetes. Nat Commun. 2023;14(1):3904. 37400433 10.1038/s41467-023-38234-w PMC10318084

[9] Wang Y, Lee D, Brellenthin AG, Sui X, Church TS, Lavie CJ, Association of muscular strength and incidence of type 2 diabetes. May Clin Proc. 2019;94(4):643–51. 10.1016/j.mayocp.2018.08.037 PMC6450733 30871784

[10] Bhasin S, Travison TG, Manini TM, Patel S, Pencina KM, Fielding RA, Sarcopenia definition: the position statements of the sarcopenia definition and outcomes consortium. J Am Geriatr Soc. 2020;68(7):1410–8. 32150289 10.1111/jgs.16372 PMC12132920

[11] McGrath R, Johnson N, Klawitter L, Mahoney S, Trautman K, Carlson C, What are the association patterns between handgrip strength and adverse health conditions? A topical review. SAGE Open Med. 2020;8:2050312120910358. 32166029 10.1177/2050312120910358 PMC7052448

[12] Manda CM, Hokimoto T, Okura T, Isoda H, Shimano H, Wagatsuma Y. Handgrip strength predicts new prediabetes cases among adults: A prospective cohort study. Prev Med Rep. 2020;17:101056. 32071846 10.1016/j.pmedr.2020.101056 PMC7016270

[13] Boonpor J, Parra-Soto S, Petermann-Rocha F, Ferrari G, Welsh P, Pell JP, Associations between grip strength and incident type 2 diabetes: findings from the UK Biobank prospective cohort study. BMJ Open Diabetes Res Care. 2021;9(1):e001865. 10.1136/bmjdrc-2020-001865 PMC8344322 34353878

[14] McGrath R, Cawthon P, Clark B, Fielding R, Lang J, Tomkinson G. Recommendations for Reducing Heterogeneity in Handgrip Strength Protocols. J Frailty Aging. 2022;11(2):143–50. 35441190 10.14283/jfa.2022.21

[15] Peterson MD, McGrath R, Zhang P, Markides KS, Al Snih S, Wong R. Muscle weakness is associated with diabetes in older Mexicans: the Mexican health and aging study. J Am Med Dir Assoc. 2016;17(10):933–8. 27450948 10.1016/j.jamda.2016.06.007 PMC5039066

[16] Brown EC, Buchan DS, Madi SA, Gordon BN, Drignei D. Grip strength cut points for diabetes risk among apparently healthy US adults. Am J Prev Med. 2020;58(6):757–65. 32273132 10.1016/j.amepre.2020.01.016

[17] Peterson MD, Zhang P, Choksi P, Markides KS, Al Snih S. Muscle weakness thresholds for prediction of diabetes in adults. Sports Med. 2016;46:619–28. 26744337 10.1007/s40279-015-0463-z PMC4863981

[18] Manini TM, Patel SM, Newman AB, Travison TG, Kiel DP, Shardell MD, Identification of sarcopenia components that discriminate slow walking speed: a pooled data analysis. J Am Geriatr Soc. 2020;68(7):1419–28. 32633834 10.1111/jgs.16524 PMC8018524

[19] Cawthon PM, Manini T, Patel SM, Newman A, Travison T, Kiel DP, Putative cut-points in sarcopenia components and incident adverse health outcomes: an SDOC analysis. J Am Geriatr Soc. 2020;68(7):1429–37. 32633824 10.1111/jgs.16517 PMC7508260

[20] Patel SM, Duchowny KA, Kiel DP, Correa-de-Araujo R, Fielding RA, Travison T, Sarcopenia Definition & Outcomes Consortium Defined Low Grip Strength in Two Cross-Sectional, Population-Based Cohorts. J Am Geriatr Soc. 2020;68(7):1438–44. 32633830 10.1111/jgs.16419

[21] Donini LM, Busetto L, Bischoff SC, Cederholm T, Ballesteros-Pomar MD, Batsis JA, Definition and diagnostic criteria for sarcopenic obesity: ESPEN and EASO consensus statement. Obes Facts. 2022;15(3):321–35. 35196654 10.1159/000521241 PMC9210010

[22] Mesinovic J, Zengin A, De Courten B, Ebeling PR, Scott D. Sarcopenia and type 2 diabetes mellitus: a bidirectional relationship. Diabetes Metab Syndr Obes. 2019;12:1057–72. 31372016 10.2147/DMSO.S186600 PMC6630094

[23] McGrath R, Tomkinson GR, Hamm JM, Juhl K, Knoll K, Parker K, The Role of Different Weakness Cut-Points for Future Cognitive Impairment in Older Americans. J Am Med Dir Assoc. 2023;24(12):1936–41. 37634549 10.1016/j.jamda.2023.07.021 PMC10840802

[24] Beaudart C, Rolland Y, Cruz-Jentoft AJ, Bauer JM, Sieber C, Cooper C, Assessment of muscle function and physical performance in daily clinical practice. Calcif Tissue Int. 2019;105(1):1–14. 30972475 10.1007/s00223-019-00545-w

[25] Fisher GG, Ryan LH. Overview of the health and retirement study and introduction to the special issue. Work Aging Retire. 2018;4(1):1–9. 29423243 10.1093/workar/wax032 PMC5798643

[26] Sonnega A, Faul JD, Ofstedal MB, Langa KM, Phillips JW, Weir DR. Cohort profile: the health and retirement study (HRS). Int J Epidemiol. 2014;43(2):576–85. 24671021 10.1093/ije/dyu067 PMC3997380

[27] HRS. Sample Sizes and Response Rates. Available from: https://hrs.isr.umich.edu/sites/default/files/bibho/sampleresponse.pdf. Accessed 2024 Jun 18.

[28] HRS. HRS Data Book. Available from: https://hrs.isr.umich.edu/about/data-book. Accessed 2024 Jun 18.

[29] Crimmins E, Guyer H, Langa K, Ofstedal MB, Wallace R, Weir D. Documentation of Physical Measures, Anthropometrics and Blood Pressure in the Health and Retirement Study. Available from: https://hrs.isr.umich.edu/sites/default/files/biblio/dr-011.pdf. Accessed 2024 Jun 18.

[30] Turvey CL, Wallace RB, Herzog R. A revised CES-D measure of depressive symptoms and a DSM-based measure of major depressive episodes in the elderly. Int Psychogeriatr. 1999;11(2):139–48. 11475428 10.1017/s1041610299005694

[31] Feng X, Croteau K, Kolt GS, Astell-Burt T. Does retirement mean more physical activity? A longitudinal study. BMC Public Health. 2016;16(1):1–7. 27439914 10.1186/s12889-016-3253-0 PMC4955250

[32] Lamarca R, Alonso J, Gomez G, Muñoz Á. Left-truncated data with age as time scale: an alternative for survival analysis in the elderly population. J Gerontol A Biol. 1998;53(5):M337–43. 10.1093/gerona/53a.5.m337 9754138

[33] Parasoglou P, Rao S, Slade JM. Declining skeletal muscle function in diabetic peripheral neuropathy. Clin Ther. 2017;39(6):1085–103. 28571613 10.1016/j.clinthera.2017.05.001 PMC5503477

[34] McGrath R. Comparing absolute handgrip strength and handgrip strength normalized to body weight in aging adults. Aging Clin Exp Res. 2019;31(12):1851–3. 30806906 10.1007/s40520-019-01126-5

[35] Albers PH, Pedersen AJ, Birk JB, Kristensen DE, Vind BF, Baba O, Human muscle fiber type–specific insulin signaling: impact of obesity and type 2 diabetes. Diabetes. 2015;64(2):485–97. 25187364 10.2337/db14-0590

[36] Taha M, AlNaam YA, Al Maqati T, Almusallam L, Altalib G, Alowfi D, Impact of muscle mass on blood glucose level. J Basic Clin Physiol Pharmacol. 2021;33(6):779–87. 34856088 10.1515/jbcpp-2021-0316

[37] CDC. Worksite Health Promotion. Available from: https://www.cdc.gov/workplacehealthpromotion/index.html. Accessed 2024 Jun 18.

[38] CDC. National Diabetes Prevention Program. Available from: https://www.cdc.gov/diabetes-prevention/?CDC_AAref_Val=https://www.cdc.gov/diabetes/prevention/index.html. Accessed 2024 Jun 18.

[39] Bauer MS, Damschroder L, Hagedorn H, Smith J, Kilbourne AM. An introduction to implementation science for the non-specialist. BMC Psychol. 2015;3(1):32. 26376626 10.1186/s40359-015-0089-9 PMC4573926

[40] Bauer MS, Kirchner J. Implementation science: What is it and why should I care? Psychiatry Res. 2020;283:112376. 31036287 10.1016/j.psychres.2019.04.025

[41] Nevill AM, Tomkinson GR, Lang JJ, Wutz W, Myers TD. How should adult handgrip strength be normalized? Allometry reveals new insights and associated reference curves. Med Sci Sports Exerc. 2022;54(1):162–8. 34431826 10.1249/MSS.0000000000002771

